# The effect of TIM1^+^ Breg cells in myocardial ischemia-reperfusion injury

**DOI:** 10.1038/s41420-025-02725-0

**Published:** 2025-10-07

**Authors:** Cong Zeng, Jianchuan Qi, Feifei Wu, Weijun Yang, Minjian Kong, Haifeng Cheng, Aiqiang Dong, Jie Han, Wei Chen, Dajin Chen, Qunjun Duan

**Affiliations:** 1 Department of Cardiology, the First Affiliated Hospital, School of Medicine, Zhejiang University, Hangzhou, Zhejiang China; 2 Department of Cardiology, Affiliated Xiaoshan Hospital, Hangzhou Normal University, Hangzhou, China; 3 Department of Cardiac Surgery, the Second Affiliated Hospital of Zhejiang University School of Medicine, Hangzhou, Zhejiang China; 4 Department of General Surgery, Sir Run Run Shaw Hospital, School of Medicine, Zhejiang University, Hangzhou, Zhejiang China; 5 Provincial Key Laboratory of Precise Diagnosis and Treatment of Abdominal Infection, Sir Run Run Shaw Hospital, School of Medicine, Zhejiang University, Zhejiang China; 6 Kidney Disease Center, the First Affiliated Hospital, school of Medicine, Zhejiang University, Hangzhou, Zhejiang China

**Keywords:** Cell biology, Immunology

## Abstract

Recent studies found that treatment with an anti-T-cell immunoglobulin mucin-1 (TIM1) monoclonal antibody (RMT1-10) regulated immune responses by inducing regulatory B cells (Bregs). However, the role of these cells in myocardial ischemia-reperfusion injury (IRI) is unknown. This study aimed to investigate the protective effect of RMT1-10 on myocardial IRI and its potential mechanism. We established a myocardial IRI model, and Triphenyl tetrazolium chloride staining, Terminal deoxynucleotidyl transferase nick-end-labeling, hematoxylin and eosin, and transmission electron microscopy were performed to examine the myocardial infarction size, myocardial cell apoptosis, and cardiomyocyte morphology and structure. The data showed that RMT1-10 could alleviate myocardial IRI, increase the number of TIM1^+^Bregs and interleukin 10 (IL-10) secretion, and regulate the expression of inflammatory factors after myocardial IRI. However, treatment with RMT1-10 and Anti-CD20 abrogated the protective effect of RMT-10. In addition, RMT1-10 treatment inhibited T cells but significantly activated Tregs after IRI, while RMT1-10 combined with Anti-CD20 abolished this effect on Tregs. Furthermore, sequencing analysis showed marked expression changes among genes related to several classical signaling pathways in response to RMT1-10. Taken together, these findings indicated that RMT1-10 could increase the number of TIM1^+^ Bregs and regulate IL-10-mediated inflammatory reactions, activate Tregs to inhibit inflammation, and might regulate the above-mentioned signaling pathways to protect against myocardial IRI.

## Introduction

Myocardial ischemia has become a leading cause of morbidity and mortality worldwide [1], causing arrhythmia, cardiac dysfunction, myocardial infarction, and even sudden death. In recent years, reperfusion techniques, such as thrombolytic therapy and first percutaneous coronary intervention, have been developed, which have significantly reduced mortality and the infarct size, and improved cardiac function [2]. However, reperfusion itself can also cause damage to the structure or function of the heart, leading to myocardial ischemia reperfusion injury (IRI) [3]. The occurrence of myocardial IRI will expand the infarct size; lead to the accumulation of inflammatory cells; seriously damage vascular endothelial function; cause obvious metabolic dysfunction, arrhythmia, and myocardial cell apoptosis; and aggravate myocardial infarction [4, 5]. Therefore, it is important to analyze the relevant molecular mechanisms of myocardial IRI and provide research ideas and a theoretical basis for the development of effective therapies.

T-cell immunoglobulin mucin-1 (TIM1) is a member of the TIM protein family, which directly couples phosphotyrosine-dependent intracellular signaling pathways and provides co-stimulatory signals for T cell activation [6]. TIM1 is a co-stimulatory molecule that is expressed on both activated CD4^+^ cells and polarized T helper 2 (Th2) cells in vitro [7, 8]. TIM1 plays an important role in effector differentiation of CD4^+^ cells and is an effective regulator of the T cell effector response [9, 10]. Its expression is much higher in all major B cell subsets than in T cells, particularly on IL-10-expressing regulatory B cells (Bregs), and is considered a marker of IL-10^+^ Bregs [11, 12]. Dysregulated IL-10^+^ TIM1^+^ Breg populations have been associated with inflammatory diseases in humans [13]. Recent studies have shown that the anti-TIM1 monoclonal antibody, RMT1-10, could increase the number of TIM1^+^ Bregs in a mouse pancreatic transplantation model, produce IL-4 and IL-10, promote the Th2 cell response, inhibit inflammation, and reduce immune rejection. RMT1-10 could also increase the secretion of cytokines by coupling TIM1 [11]; however, when TIM1 is defective or mutated in B cells, IL-10 is defective, and pro-inflammatory cytokine expression increases. In a mouse liver IRI model, RMT1-10 treatment significantly inhibited hepatocyte apoptosis [14]. In addition, in cerebral IRI, RMT1-10 treatment could also effectively inhibit the inflammatory response, thereby reducing brain injury [15]. However, the effect of RMT1-10 in myocardial IRI was not investigated.

In the present study, using TIM1^+^ Bregs as the target, we investigated the protective effect of RMT1-10 on myocardial IRI using an in vitro TIM1^+^ Breg cell co-culture system and a myocardial IRI model in vivo. We further revealed the mechanism of RMT1-10 activation of TIM1^+^ Bregs. These data not only enrich the molecular mechanism theory of the RMT1-10-mediated increase in TIM1^+^ Breg cell numbers and IL-10 secretion, but also provide a theoretical basis for TIM1 as a novel target for the treatment of myocardial IRI.

## Results

### RMT1-10 alleviates myocardial IRI

**Fig. 1:**
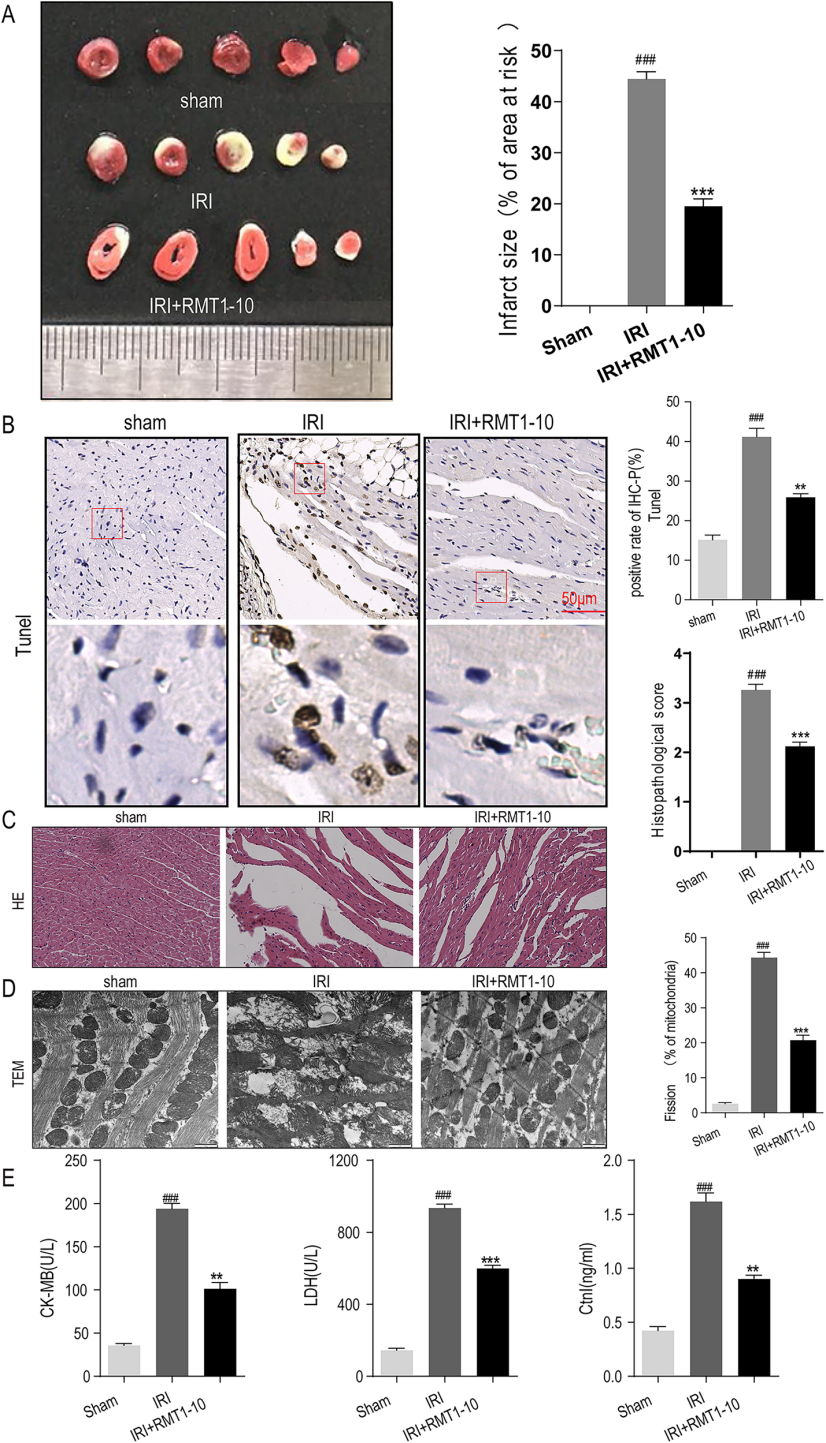
RMT1-10 alleviates myocardial IRI. **A** TTC staining of the changes in the myocardial infarction volume in the different groups. **B**, **C** TUNEL and HE staining to determine myocardial cell apoptosis and the changes in cardiomyocyte morphology. **D** Electron microscopy detection of cardiomyocyte structures in the Sham, IRI, and IRI + RMT1-10 groups. ###P < 0.001 *vs*. sham, ***P < 0.001 *vs*. IRI. **E** ELISA, LDH assays, and cTnT/I detection for the expression of CK-MB, LDH, and cTnT/I in serum. ###P < 0.001 *vs*. Sham, **P < 0.01,***P < 0.001 *vs*. IRI.

To determine the effect of RMT1-10 on myocardial IRI, we established a C57BL/6J mouse myocardial IRI model, as shown as in Fig. 1A. Triphenyl tetrazolium chloride (TTC) staining was used to observe the changes in the myocardial infarction volume in the mice, showing that the myocardial infarction size of the IRI group was larger than that of the Sham operation group, while RMT1-10 treatment could reduce the myocardial infarction size. Terminal deoxynucleotidyl transferase nick-end-labeling (TUNEL) staining also showed that the number of apoptotic cells was increased after IRI treatment in comparison with that in the Sham group; however, RMT1-10 treatment reversed the increase in apoptotic cells induced by IRI (Fig. 1B). Furthermore, hematoxylin and eosin (HE) analysis showed that the mice in the Sham group had normal cardiomyocyte morphology, whereas the IRI group had severe deformation, discontinuity, or necrosis of the tissue structure. RMT1-10 treatment could reduce this IRI-induced damage (Fig. 1C). The results of transmission electron microscopy (TEM) analysis showed that the myocardial cells in the Sham group had a clear structure, obvious myofibrils, regular arrangement, clear hierarchy, and normal and complete mitochondria. In the IRI group, the myofibrillar arrangement was irregular, myofilaments were dissolved, the mitochondrial volume increased, and rupture, necrosis, myocardial cell vacuolation, and swelling were observed. RMT1-10 treatment could reduce these pathological changes induced by IRI (Fig. 1D). Detection of myocardial injury markers (creatine phosphokinase-MB (CK-MB); lactate dehydrogenase (LDH), and the cardiac troponin T/I ratio (cTnT/I)) in serum indicated that LDH, the activity of CK-MB, and cTnT/I were increased in the IRI group, whereas RMT1-10 treatment could reduce them (Fig. 1E).

### RMT1-10 increased the number of TIM1^+^Bregs and regulated the expression of inflammatory factors after myocardial IRI-induction

**Fig. 2:**
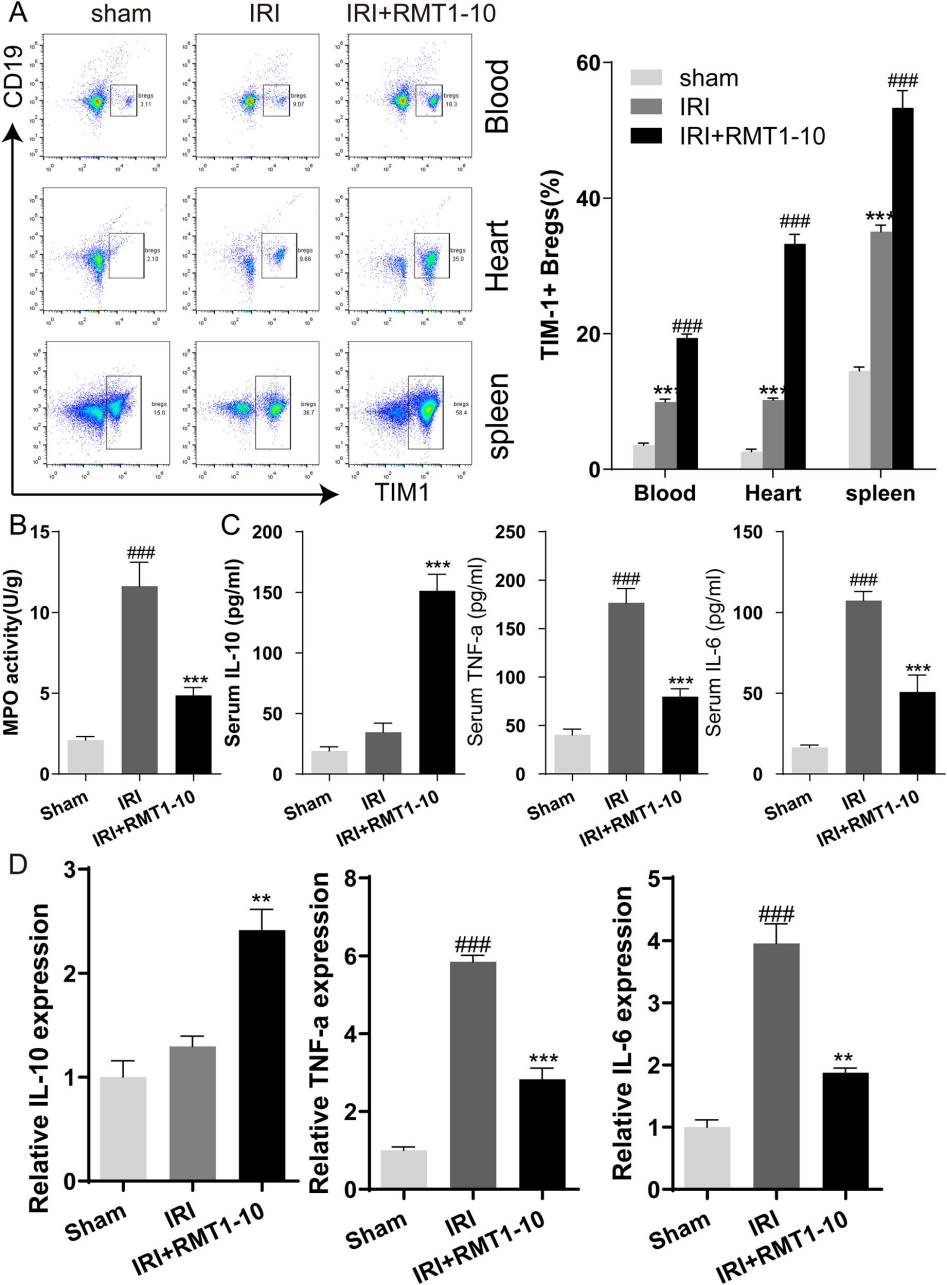
RMT1-10 increased the number of TIM1^+^ Bregs and regulated the expression of inflammatory factors after myocardial IRI-induction. **A** Flow cytometry analysis of the changes of TIM1^+^ Bregs (TIM1^+^ CD19^+^) in the different treatment groups (Sham, IRI, and IRI + RMT1-10) in the spleen, heart, and blood. ***P < 0.001 *vs*. Sham, ###P < 0.001 *vs*. IRI. **B** The MPO activity in the different groups. ###P < 0.001 *vs*. Sham, ***P < 0.001 *vs*. IRI. **C** ELISA determination of the serum levels of IL-10, TNFα, and IL-6 in the different groups. **D** qRT-PCR was used to determine the expression levels of *Il-10*, *Tnfa*, and *Il-6* in the different groups of spleen cells (with red blood cells removed).

To further analyze the effect of RMT1-10 on inflammatory factors, peripheral blood samples, myocardial immune cells, and spleen cells (after removing red blood cells) were acquired from the different treatment groups. We then examined the changes in the numbers of TIM1^+^ Bregs (TIM1^+^ CD19^+^) using flow cytometry. The number of TIM1^+^ Bregs increased after myocardial IRI in the blood, heart, and spleen; however, a larger increase was observed after RMT1-10 intervention in the IRI model mice in comparison with that in the IRI group (Fig. 2A). The activity of myeloperoxidase (MPO), used to assess the degree of neutrophil infiltration and inflammatory reaction in the mice, was increased in the IRI group, but was reduced after RMT1-10 treatment (Fig. 2B). Enzyme-linked immunosorbent assay (ELISA) and quantitative real-time reverse transcription PCR (qRT-PCR) were used to further analyze the expression levels of interleukin (IL)-10, tumor necrosis factor alpha (TNFα), and IL- 6 in peripheral blood serum and spleen cells, respectively. RMT1-10 treatment enhanced the expression of anti-inflammatory factors, such as IL-10, and reduced the expression of pro-inflammatory factors, such as TNFα and IL-6, compared with those in the IRI group (Fig. 2C, D).

The diagram of establishing the model and the gating strategies for TIM1^+^ Bregs in the spleen, heart, and blood are shown in Supplementary Fig. 1A, B. Next, we checked the relationship between TIM1 expression and myocardial IRI. After myocardial IRI, the level of TIM1 in cardiomyocytes increased slightly, while RMT1-10 treatment further promoted the expression of TIM1 and reduced the apoptosis and necrosis of cardiomyocytes, thus playing a protective role against myocardial IRI (Supplementary Fig. 1C).

### Anti-CD20 alleviates the protective effect of RMT1-10 in myocardial IRI

**Fig. 3:**
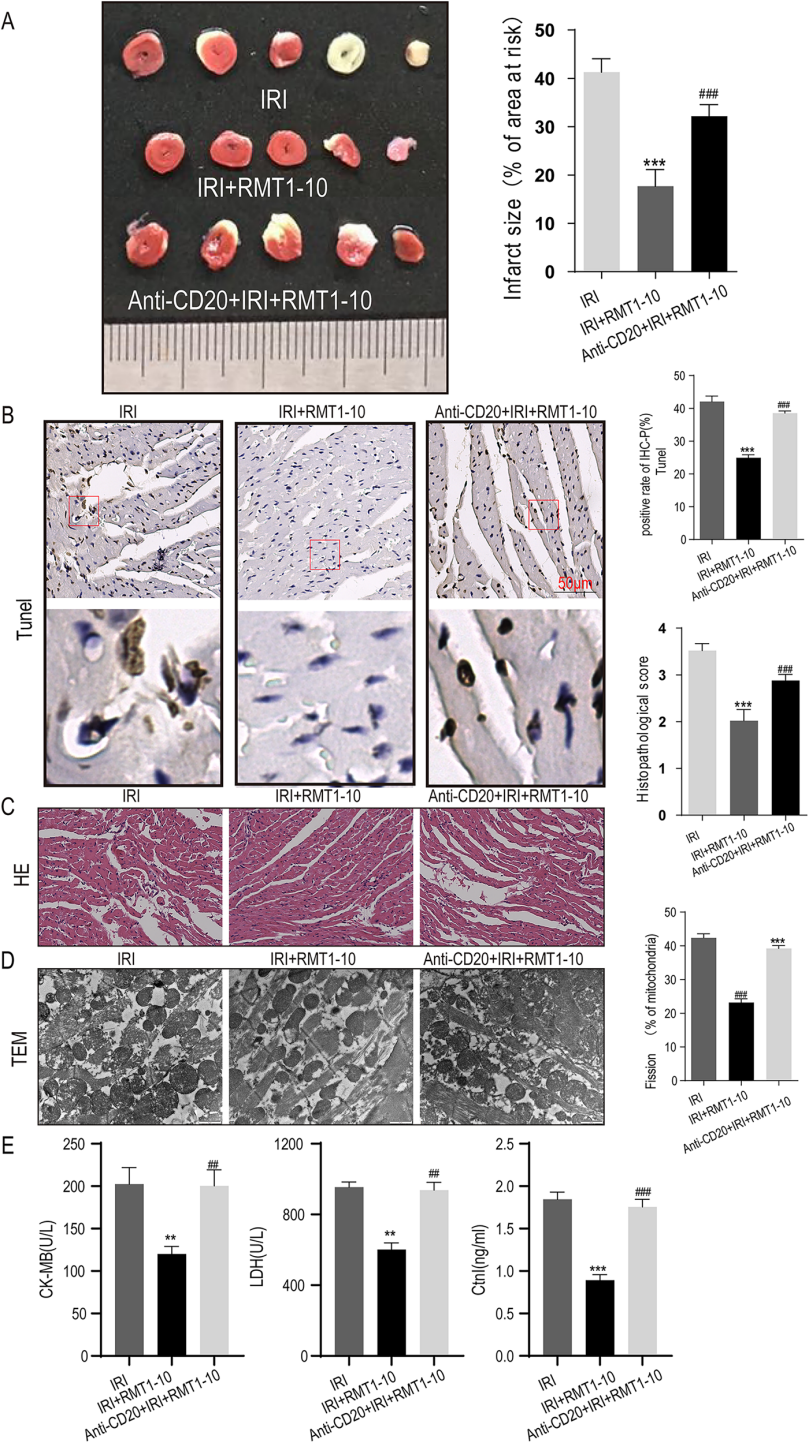
Anti-CD20 reverses the protective effect of RMT1-10 in myocardial IRI. **A** The myocardial infarction volume, as determined by TTC in the IRI, IRI + RMT1-10, and Anti-CD20 + IRI + RMT1-10 groups. **B**, **C** TUNEL and HE staining for myocardial cell apoptosis and changes in cardiomyocyte morphology in the IRI, IRI + RMT1-10, and Anti-CD20 + IRI + RMT1-10 groups. **D** Electron microscopy analysis of the cardiomyocyte structure in the IRI, IRI + RMT1-10, and Anti-CD20 + IRI + RMT1-10 groups. ***P < 0.001 *vs*. IRI, ###P < 0.001 *vs*. IRI + RMT1-10. **E** ELISA, LDH assays, and cTnT/I detection of the levels of CK-MB, LDH, and cTnT/I in serum. **P < 0.01,***P < 0.001 *vs*. IRI, ###P < 0.001 *vs*. IRI + RMT1-10.

TIM1 is expressed on Bregs that express IL-10 in all major B cell subsets and is considered a marker of IL-10^+^Bregs [16]. To explore the protective effect of TIM1^+^ Bregs on myocardial IRI, we established a B-cell-specific defective mouse model by treating the mice with an Anti-CD20 antibody, then established the myocardial IRI model in these mice, and treated them with RMT1-10 to further observe the extent of myocardial injury in the different treatment groups. TTC staining showed that the myocardial infarction volume increased after treatment with Anti-CD20 combined with RMT1-10 (Fig. 3A). TUNEL and HE staining also proved that combined Anti-CD20 and RMT1-10 treatment increased the number of apoptotic myocardial cells and cardiomyocyte injury, respectively (Fig. 3B, C). Myocardial ultrastructure was observed using TEM, which showed that mitochondrial fission was increased in the Anti-CD20 + IRI + RMT1-10 group (Fig. 3D). Moreover, we found that LDH, the activity of CK-MB, and cTnT/I were increased in the Anti-CD20 + IRI + RMT1-10 group in comparison with those in the IRI + RMT1-10 group (Fig. 3E). The above data demonstrated that the protective effect of RMT-10 vanished after treatment with Anti-CD-20.

### Anti-CD20 reduces the number of TIM1^+^ Bregs and enhances the expression of inflammatory factors after co-treatment with RMT1-10 in myocardial IRI

**Fig. 4:**
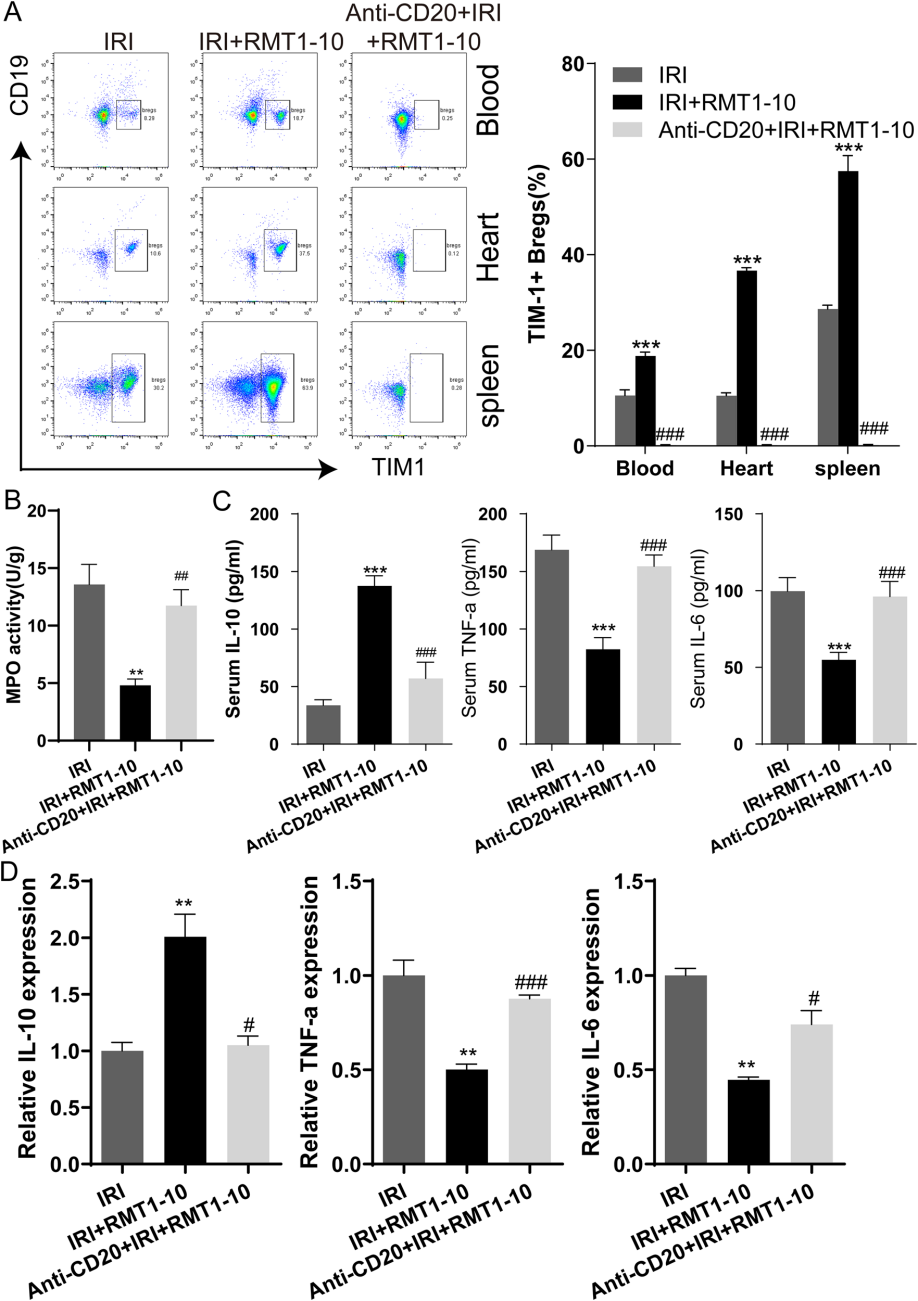
Anti-CD20 reduces the number of TIM1^+^ Bregs and enhances the expression of inflammatory factors after co-treatment with RMT1-10 in myocardial IRI. **A** The changes in the number TIM1^+^ Bregs (TIM1^+^ CD19^+^) in the different treatment groups as determined by Flow cytometry analysis. ***P < 0.001 *vs*. IRI, ###P < 0.001 *vs*. IRI + RMT1-10. **B** Detection of MPO activity in the different groups. **P < 0.01 *vs*. IRI, ##P < 0.01 *vs*. IRI + RMT1-10. **C** C. ELISA determination of the serum levels of IL-10, TNFα, and IL-6 in the different groups. ***P < 0.001 *vs*. IRI, ###P < 0.001 *vs*. IRI + RMT1-10. **D** qRT-PCR was used to determine the expression levels of *Il-10*, *Tnfa*, and *Il-6* in the different groups of spleen cells (with red blood cells removed). **P < 0.01 *vs*. IRI, #P < 0.05, ###P < 0.001 *vs*. IRI + RMT1-10.

We then performed detailed phenotypic and functional characterization of these TIM1^+^ Bregs. As shown in Fig. 4A, the number of TIM1^+^ Bregs was reduced in the blood, heart, and spleen after myocardial IRI in the Anti-CD20 + IRI + RMT1-10 group in comparison with those in the IRI + RMT1-10 group. The activity of MPO was downregulated after RMT1-10 treatment under IRI conditions; however, it increased after co-treatment with Anti-CD20 and RMT1-10 (Fig. 4B). Compared with those in the IRI + RMT1-10 group, the level of IL-10 decreased, and TNFα and IL-6 levels increased in the Anti-CD20 + IRI + RMT1-10 group (Fig. 4C, D).

### RMT1-10 treatment increases TIM1^+^ Bregs and IL-10 secretion in vitro

**Fig. 5:**
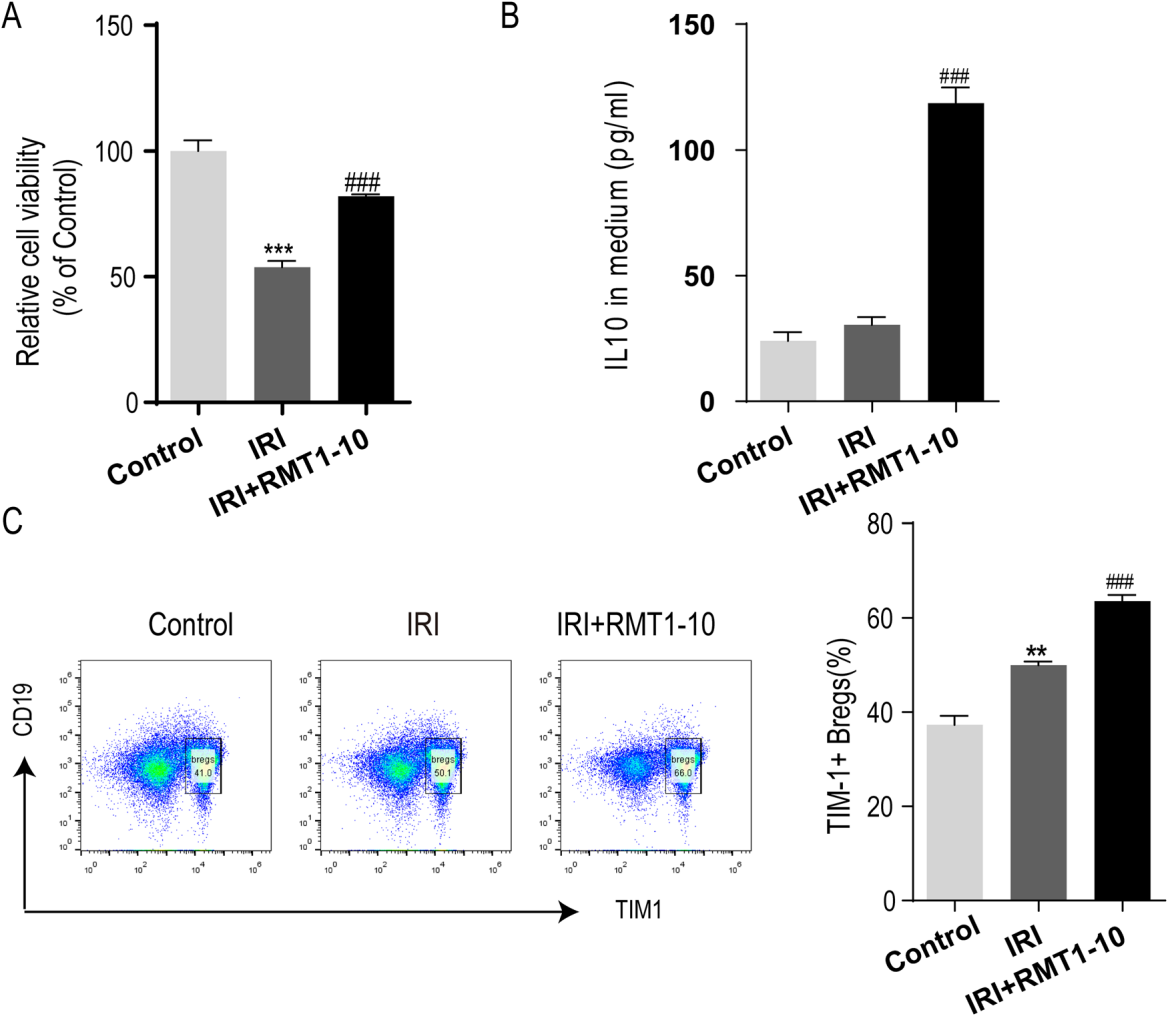
RMT1-10 treatment increases TIM1^+^ Bregs and IL-10 secretion in vitro. **A** CCK-8 analysis of myocardial cell viability in the Control, IRI, and IRI + RMT1-10 groups. ***P < 0.001 *vs*. Control, ###P < 0.001 *vs*. IRI. **B** The levels of IL-10 in the supernatant, as detected by ELISA. **C** Flow cytometry detection of TIM1^+^ Bregs (TIM1^+^ CD19^+^). **P < 0.01 *vs*. Control, ###P < 0.001 *vs*. IRI.

The above data preliminary confirmed that RMT1-10 could alleviate myocardial IRI by increasing the number of TIM1^+^ Bregs and regulating inflammatory factors in vivo. To further reveal the effect of RMT1-10 in vitro, we isolated mouse spleen B cells, T cells, and primary cardiomyocytes (sham and IRI) using magnetic beads and co-cultured them with RMT1-10. We then observed the changes in cardiomyocyte activity, IL-10 expression, and the number of TIM1^+^ Bregs (TIM1^+^ CD19^+^). The results showed that cardiomyocyte cell viability was reduced after IRI, while RMT1-10 treatment could significantly increase it (Fig. 5A). The levels of IL-10 and TIM1^+^ Bregs increased transiently after myocardial IRI, whereas RMT1-10 intervention significantly increased them (Fig. 5B, C). These results indicated that RMT1-10 could promote the secretion of IL-10 by TIM1^+^ Breg cells.

The homologous interaction between Breg cells and T cells is considered to control the induction of Treg cells [17–19]. After RMT1-10 treatment, there was a significant increase in Treg cells (CD4^+^CD25^+^Foxp3^+^) (Supplementary Fig. 2A). When the B cells were removed by treatment with Anti-CD20 antibodies, RMT1-10 treatment could not increase the number of Treg cells (CD4^+^CD25^+^Foxp3^+^) after IRI (Supplementary Fig. 2B). TIM1^+^ Bregs were co-cultured with CD4^+^ naive T cells and CD8^+^ naive T cells, separately. Flow cytometry detection of the differentiation of Th1 (CD4^+^ T and CD8^+^ T) subsets showed that T cells differentiated significantly into CD4^+^ cells (Supplementary Fig. 2C). The above data indicated that TIM1^+^ Bregs could promote the secretion of immunomodulatory cytokine IL-10, thus further regulating Tregs, inhibiting the inflammatory response, and alleviating myocardial IRI.

### The molecular mechanism by which RMT1-10 promotes the proliferation of TIM1^+^ Bregs and the secretion of IL-10

**Fig. 6:**
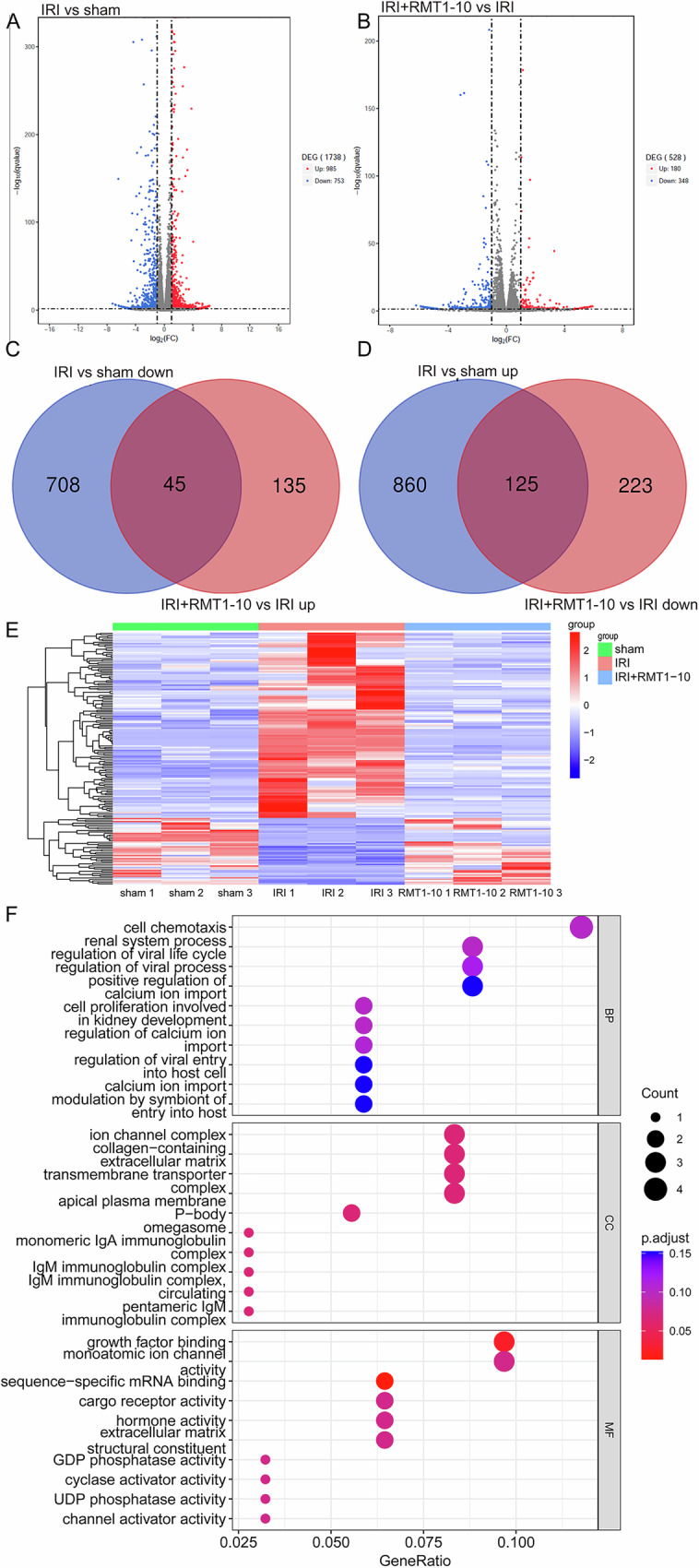
The molecular mechanism by which RMT1-10 promotes the proliferation of TIM1+ Bregs and the secretion of IL-10. **A**, **B** Differentially expressed genes among the different groups, as analyzed using a volcano map. **C**, **D** Venn diagrams of the upregulated and downregulated genes among the sham, IRI, and IRI + RMT1-10 groups. **E** Heat map analysis of the differentially expressed genes. **F** GO enrichment analysis of the differentially expressed genes.

To further investigate the signaling pathways through which RMT1-10 increases the number of TIM1^+^ Bregs and the secretion of IL-10, we carried out mRNA sequencing analysis in the Sham, myocardial IRI, and RMT1-10 + IRI groups. Subsequently, a volcano plot showed the upregulated and downregulated genes map between the sham and IRI groups, and between the IRI and IRI + RMT1-10 groups (Fig. 6A, B). Intersection analysis of the downregulated genes between the Sham and IRI groups and the upregulated genes between the IRI and IRI + RMT1-10 groups identified 45 genes. Intersection analysis of the upregulated genes between the Sham and IRI groups and the downregulated genes between the IRI and IRI + RMT1-10 groups identified 125 genes (Fig. 6C, D). This information was processed in a heat map (Fig. 6E).

We also provided a PCA graph to demonstrate that the differences between groups are greater than those within groups (Supplementary Fig. 4).

### RMT1-10 protects myocardial IRI via the EGFR pathway

**Fig. 7:**
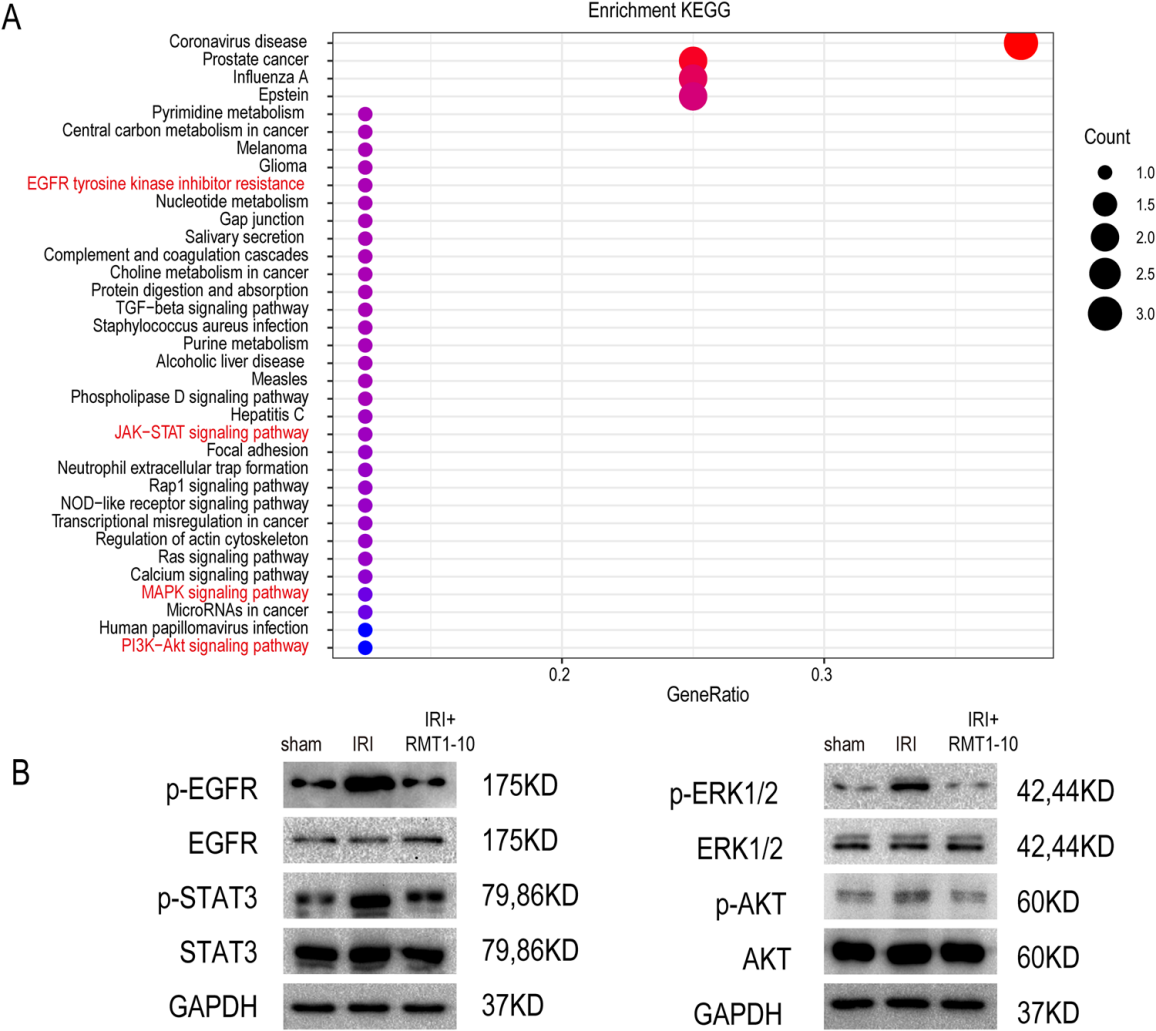
RMT1-10 protects myocardia against IRI via the EGFR pathway. **A** KEGG enrichment analysis of the differentially expressed genes. **B** Western Blotting detection of the levels of EGFR, STAT3, ERK1/2, and AKT.

We further used Kyoto Encyclopedia of Genes and Genomes (KEGG) enrichment analysis to analyze the above differentially expressed genes, which showed that they were enriched in many signaling pathways. We selected several classical signaling pathways, such as the epidermal growth factor receptor (EGFR) signaling pathway, the Janus kinase (JAK)-signal transducer and activator of transcription (STAT) signal pathway, the mitogen activated protein kinase (MAPK) signaling pathways, and the phosphatidylinositol-4,5-bisphosphate 3-kinase (PI3K)-protein kinase B (AKT) signaling pathway for further analysis (Fig. 7A, Supplementary Fig. 3). We detected related protein levels in the Sham, IRI, and IRI + RMT1-10 groups, which showed that phosphorylated ((p)-EGFR), p-STAT3, p-extracellular regulated kinase 1/2 (ERK1/2), and p-AKT levels were upregulated after IRI, while these indicator proteins were downregulated after RMT1-10 treatment (Fig. 7B).

## Discussion

Myocardial IRI is related to apoptosis and necrosis of cardiomyocytes, which reduces the chance of a cure after thrombolytic therapy, and involves inflammation, oxidative stress, calcium overload, and other factors [20, 21]. In this study, we investigated the protective effect of TIM1 on myocardial IRI. TIM1 was found recently to be predominantly expressed on Bregs [11, 22]. Binding of TIM1 by low-affinity RMT1-10 promoted immune tolerance via IL-10-expressing B cells, and treatment with RMT1-10 also increased TIM1^+^ Breg numbers and the percentage of TIM1^+^ Bregs expressing IL-10 and IL-4, thereby preserving Tregs but inhibiting CD4^+^ Th1 cell expansion [11, 23]. TIM1 plays an important role in regulating the function of Bregs, inhibiting the inflammatory response, and maintaining self-tolerance. However, the role of TIM1^+^ Bregs in myocardial IRI has not been investigated. Our data showed that there was a transient increase in TIM1 expression in myocardial tissue at 24 h after myocardial IRI, and the number of TIM1^+^ positive cells increased, mainly B cells. Furthermore, the expression of TIM1 in Bregs was significantly upregulated after RMT1-10 treatment in a mouse model of myocardial IRI. These results suggested that the activation of TIM1^+^ Bregs could protect against myocardial IRI in mice, and the effect of RMT1-10 could increase the number of TIM1^+^ Bregs, thereby reducing myocardial IRI.

Bregs are associated with suppressing excessive inflammation [24, 25]. They can inhibit the differentiation of Th1 and Th17 cells by inhibiting the production of pro-inflammatory cytokines in dendritic cells [26]. In addition to expressing IL-10, Bregs also express other immunomodulatory cytokines, including transforming growth factor-β (TGFβ) and IL-35 [27, 28]. By producing TGFβ, lipopolysaccharide (LPS)-activated B cells can induce CD4^+^ apoptosis and non-reactive CD8^+^ T cells [29]. In addition, IL-35 is associated with Breg-mediated immunosuppression and can improve immune tolerance by inducing Treg proliferation and inhibiting the Th17 response [30, 31]. It has also been reported that human Bregs are crucial in maintaining invariant natural killer T (iNKT) cell homeostasis [32, 33]. Therefore, Bregs play many roles in suppressing the immune response and have the ability to target multiple immune system cells, thus playing a pleiotropic role. Our data showed that IL-10 secretion was slightly upregulated after myocardial IRI in mice, but the difference was not obvious; however, RMT1-10 treatment significantly upregulated the expression of IL-10. These results suggested that RMT1-10 could promote IL-10 secretion from TIM1^+^ Bregs.

Deficiency of IL-10^+^ Bregs would further aggravate the development of various inflammatory immune diseases [28]. Transgenic mice that lacked B cells (specifically B cells that produce IL-10) show chronic inflammation because of defects in the development and function of Bregs [34, 35]. In mice and humans, Bregs support the regulation of various phenotypes by distorting T cell differentiation. The importance of B cells in maintaining Tregs can be inferred from earlier studies that showed that Tregs were significantly reduced in B-cell-deficient mice [36]. Mice with B-cell-specific IL-10 deficiency also exhibited Treg cell deficiency, which was associated with the growth of pro-inflammatory T cells after autoimmune induction [37]. Therefore, we further detected immune cells of mice after myocardial IRI, which showed that the number of Tregs (CD4^+^CD25^+^Foxp3^+^) increased after myocardial IRI + RMT1-10, while after treatment with Anti-CD20 combined with RMT1-10, the percentage of Tregs (CD4^+^CD25^+^Foxp3^+^) was reduced.

RMT1-10 can upregulate the number of TIM1^+^ Bregs and increase the secretion of cytokines by binding to TIM1; however, when TIM1 is defective or mutated in B cells, IL-10 will be defective, and pro-inflammatory cytokine expression will increase [11, 38]. We also found that in the IRI model, after RMT1-10 treatment, the EGFR signaling pathway, JAK-STAT signal pathway, MAPK signaling pathways, p-EGFR in the PI3K-AKT signaling pathway, p-STAT3, p-ERK1/2, and p-AKT were significantly downregulated. Thus, RMT1-10 is likely to act via the EGFR JAK/STAT/MAPK/PI3K-AKT signaling pathways to promote mature B cells to increase the number of TIM1^+^ Breg and the secretion of IL-10 to regulate inflammation, thus protecting against myocardial IRI. However, the specific pathway that plays a leading role still needs to be further identified in future studies. To directly test causality, we have initiated follow-up studies using Pharmacological inhibitors (e.g., AG1478 for EGFR, Stattic for STAT3) to determine if RMT1-10’s effects are abolished; cell-specific knockout models (e.g., AAV9-Cre-mediated STAT3 deletion in cardiomyocytes). We will report these results in a future publication focused on mechanistic dissection. Although our data support TIM1⁺ Bregs as a primary target of RMT1-10, we cannot exclude potential modulation of other TIM1⁺ immune populations. Future studies incorporating comprehensive immune profiling will clarify the selectivity of RMT1-10 across cell types. This will be a key focus of our future work, including: Multi-parametric flow cytometry to dissect TIM1⁺ cell subsets and adoptive transfer experiments of TIM1⁺ Bregs versus TIM1⁺ T cells to compare their functional roles.

## Conclusions

Our findings showed that RMT1-10 could protect against myocardial IRI, possibly by regulating the EGFR/JAK/STAT/MAPK/PI3K-AKT signaling pathways. These results suggest strategies to develop TIM1^+^ Breg cellular therapy and identify potential therapeutic targets to modulate TIM1^+^ Breg function in myocardial IRI.

## Materials and methods

### Animals

C57BL/6J mice (6–8 weeks old, Male) were purchased from the Charles River Laboratory (Beijing, China). All mice were housed in an environmentally controlled room under a 12 h light/dark cycle with *ad libitum* access to food and water.

### Myocardial IRI model

A total of 24 C57BL/6J mice were randomly divided into four groups (n = 6): Sham, IRI, IRI + RMT1-10 (500 µg anti-mouse TIM1 (BioXCell, Lebanon, NH, USA; RMT1-10, i.p. on day 1 before myocardial IRI, and 300 µg on days 0 and 1)), IRI + RMT1-10+ Anti-CD20 (BioXCell, 250 μg/200 μl PBS); Sterile anti-mouse CD20 and isotype control monoclonal antibodies (mAbs) were injected through lateral tail veins on day 14 and 7 before myocardial IRI. First, the mice were anesthetized using 3% pentobarbital sodium injection, the surgical area was disinfected, the skin was cut between the third and fourth ribs on the left side of the sternum, and the heart was exposed. Next, a 6-0 suture needle was applied at 1 mm below the lower edge of the atrial ear through the surface of the heart, and the anterior descending branch of the left coronary artery (LAD) was then subjected to pressure ligation. Electrocardiography (ECG) showed that the ST segment continued to be elevated, indicating successful induction of myocardial ischemia. The thoracic cavity was closed using a simple suture. The thoracic cavity was opened again after 30 min of ischemia, and the ligature line was cut. The surface of the heart turned from pale to red, and the elevated part of the ST segment on ECG returned to the baseline, indicating successful reperfusion. The tracheal intubation was pulled out after the mouse awoke, and the mouse was placed in a thermal blanket overnight. At 2 weeks post-surgery, the mice were killed by intraperitoneal injection of 2% isoflurane. Their hearts were removed and processed for further experiments. All histopathological analyses (HE, TUNEL, and TEM) were performed specifically in the infarct zone.

### Primary cardiomyocyte extraction and culture

The heart was removed from the mature C57BL6/J mice. The myocardium was snipped and digested until the myocardium became translucent, then the digestion was terminated. The resulting cardiomyocyte suspension was centrifuged, the supernatant was discarded, the cells were washed with Dulbecco’s modified Eagle’s medium (DMEM)-F12 and 15% FBS Brdu (fetal bovine serum-bromodeoxyuridine), filtered into a petri dish, and cultured in an incubator for 2 h. Subsequently, fibroblasts were removed by differential adhesion separation to obtain cardiomyocytes.

### Triphenyl tetrazolium chloride (TTC) staining

The myocardial tissue sections were obtained from the mid-left ventricular (LV) level (papillary muscle plane) and apical third, then cut transversely into 2 mm thick slices. Slices were incubated at 1% TTC (Solarbio Life Science, Beijing, China) at 37 °C for 10 min to identify non-infarct and infarct areas.

### Terminal deoxynucleotidyl transferase nick-end-labeling (TUNEL) staining

Myocardial tissue was cut into 0.5 cm^3^ pieces and fixed using 4% paraformaldehyde phosphate. The slices at a thickness of 10 μm were prepared by routine dehydration, wax dipping, and embedding. The percentage of apoptotic cells was determined using an in situ cell death detection kit (TUNEL; Roche, Basel, Switzerland) following the supplier’s protocol. The apoptotic cells were observed under a light microscope. Normal myocardial nuclei appeared blue, and apoptotic nuclei appeared brown. The apoptosis rate was calculated from not less than 500 myocardial nuclei.

### Transmission electron microscopy (TEM)

After IRI, the heart was removed, and samples were taken from the free wall of the left ventricle using a double-sided blade. The ventricular muscle was cut into small pieces (1 mm^3^) and fixed with glutaraldehyde phosphate buffer at 4 °C for 24 h. Ultrathin sections of 50–70 nm were prepared by routine dehydration, immersion, embedding, and staining. The ultrastructure of the cardiomyocytes was observed under a transmission electron microscope.

### Hematoxylin and eosin (HE) staining and Immunohistochemical analysis

The paraffin-embedded sections on slides were deparaffinized and hydrated using an ethanol gradient. For the HE analysis, the sections were stained with hematoxylin for 5 min and subsequently with eosin for 2 min. For the immunohistochemical analysis, the sections were stained with anti-TIM1 antibody (1:500, Abcam, Cambridge, MA, USA (ab15580)) overnight at 4 °C. Then, the sections were reacted with horseradish peroxidase (HRP) and detected using a Diaminobenzidine (DAB) substrate kit (Abcam, ab64238). Finally, the sections were observed under a light microscope.

### Enzyme-linked immunosorbent assay (ELISA)

The expression of myosin and creatine kinase isoenzyme (CK-MB) was detected using ELISA, and the ratio of cardiac troponin to troponin I (cTnT/I) and Myeloperoxidase (MPO) was determined using cTnT/I and MPO detection Kits, following the supplier’s protocol.

### Cytotoxicity lactate dehydrogenase (LDH) analysis

A Cytotoxicity LDH Assay Kit-WST (Dojindo, Kumamoto, Japan) was used to determine cytotoxicity based on LDH released by damaged cells, following the supplier’s protocol.

### Co-culture of B cells, T cells, and primary cardiomyocytes

CD4^+^ naïve T (CD8^+^ naïve T) and TIM1^+^ Breg (TIM1^+^ CD19^+^ B cells) were flow sorted from the peripheral blood mononuclear cells (PBMCs) of mice. CD4^+^ naïve T (CD8^+^ naïve T) were co-stimulated with anti-CD3 (5 µg/ml)/anti-CD28 (5 µg/ml) antibodies (1 h), and TIM1^+^ CD19^+^ B cells were stimulated with anti-IL-10 neutralizing antibodies (10 µg/ml) or/and anti-TGF-β (10 µg/ml) for 3 days. Then, these two types of cells were placed in the upper chamber for co-culture. Cardiomyocytes were isolated and seeded in the lower chamber of a 24-well plate at a density of 5 × 10⁴ cells per well for adherent culture. The cells were treated with TIM1 monoclonal antibody (RMT1-10). After treatment, cells were harvested from the lower chamber, and cardiomyocyte activity was analyzed.

### Isolation of immune cells

T cells (CD4^+^ naïve T and CD8^+^ naïve T cells) and B cells were isolated from mouse splenocytes using a Dynabeads Untouched Mouse CD4 Cell Isolation Kit, a Dynabeads Untouched™ Mouse CD8 Cell Isolation Kit, and a Dynabeads® Mouse CD43 Untouched™ B Cell Isolation Kit, respectively (Miltenyi Biotec, Bergisch Gladbach, Germany).

### Flow cytometry analysis

The percent of TIM-^+^ Bregs (TIM1^+^ CD19^+^) and Tregs (CD4^+^ CD25^+^ Foxp3^+^) was determined using Flow cytometry analysis. Isolated cells were treated with anti-TIM1, anti-CD19, anti-CD4, anti-CD25, anti-forkhead box O3 (Foxp3) antibodies. The data were acquired using a Cytek Aurora 3000 spectral flow cytometer (Cytek, Fremont, CA, USA) and analyzed using FlowJo software v10.7.1 (Treestar, Ashland, OR, USA).

### Cell counting kit 8 (CCK-8) analysis

Primary myocardial cells were seeded into 96-well plates at a density of 3 × 10^3^ cells/well. Cell viability was examined with a CCK-8 assay (Dojindo). Briefly, 10 μL of CCK-8 solution was added to each well and incubated for 3 h. An MRX II microplate reader (Dynex Technologies, Chantilly, VA, USA) was used to determine the absorbance at 450 nm.

### Western blotting analysis

First, different groups of cells were treated using cell lysis buffer, and 40 μg protein were separated using 10% sodium dodecyl sulfate-polyacrylamide gel electrophoresis (SDS-PAGE), followed by transfer of the separated protein onto polyvinylidene fluoride (PVDF) membranes (Millipore, Billerica, MA, USA). The membranes were blocked using Tris-buffered saline (TBS) with 0.1% Tween 20 (TBS-T) containing 5% bovine serum albumin for 2 h at 37 °C. The membranes were washed three times with TBS-T, followed by overnight incubation at 4 °C with primary antibodies recognizing epidermal growth factor receptor (EGFR), signal transducer and activator of transcription 3 (STAT3), extracellular regulated kinase 1/2 (ERK1/2), and protein kinase B (AKT) (diluted 1:1000 in TBS-T). The next day, the membranes were incubated with HRP-conjugated secondary antibodies for 2 h at 37 °C. Glyceraldehyde-3-phosphate dehydrogenase (GADPH) was detected as an internal control. The protein bands were detected using chemiluminescence (GE Healthcare, Piscataway, NJ, USA) and were quantified based on the optical density of each band using Image J (NIH, Bethesda, MD, USA).

### Quantitative real-time reverse transcription PCR (qRT-PCR)

RNA from all samples was extracted using TRIzol (Thermo Fisher Scientific, Waltham, MA, USA), which was reverse-transcribed into cDNA using a Prime-Script RT reagent kit (Takara Biotechnology, Dalian, China). The qPCR step of the qRT-PCR protocol used the cDNA as the template and the 2× SYBR Green PCR Master Mix (Takara Biotechnology) following the supplier’s guidelines. Real-time PCR was performed using an ABI 7500 real-time PCR system (Applied Biosystems, Foster City, CA, USA). Relative gene expression levels were analyzed using the comparative 2^−ΔΔCt^ method. All the experiments were repeated at least three times.

#### *Il-10*

Forward: 5′-GAAGACCCTCAGGATGCGGC -3′;

Reverse: 5′-AAGAGACCCGACACCGGACA -3′

#### *Tnfa*

Forward: 5′- GCCAACCAGGCAGGTTCTGT -3′;

Reverse: 5′- TAGGCACCGCCTGGAGTTCT -3′

#### *Il-6*

Forward: 5′- GCCCTCTGGCGGAGCTATTG -3′;

Reverse: 5′- AAGGCCGTGGTTGTCACCAG -3′

### Sequencing analysis

Splenic B cell mRNA was sequenced by NOVO GENE Co., Ltd. (Beijing, China).

### Gene ontology (GO) and Kyoto Encyclopedia of Genes and Genomes (KEGG) pathway enrichment analysis

The differentially expressed genes among the groups were subjected to GO enrichment analysis to clarify molecular functions and biological processes, and KEGG pathway enrichment analysis to predict key pathways.

### Statistical analysis

Experimental data are expressed as the mean ± standard deviation (SD). Comparisons between two groups were carried out using Student’s t-test or one-way analysis of variance, followed by the Tukey post-hoc test comparison for multiple groups. The statistical analyses were carried out using GraphPad Prism 9.0 software (GraphPad Software, San Diego, CA, USA). Statistical significance was accepted at P < 0.05.

## Supplementary information

Supplementary Figure legends

Original data 1

Original data 2

Supplement Figure 1

Supplement Figure 2

Supplement Figure 3

Supplement Figure 4

## Data Availability

We declare that all data support the conclusions of the study, and all raw data will be made available upon request.
