# Supplementary material for: The effect of TIM1+ Breg cells in myocardial ischemia-reperfusion injury
Source: Cell Death Discov. 2025 Oct 7;11:453. doi: 10.1038/s41420-025-02725-0 (PMC12504465; doi:10.1038/s41420-025-02725-0)
Supplement: Supplementary file 1 — Supplementary Figure legends [file 41420_2025_2725_MOESM1_ESM.docx]

**Supplementary Figures**

**Figure S1**

A. Diagram of establishing the co-culture model. Anti-CD20, Anti-CD20 with RMT1‑10, or Normal Saline was administered to wild-type mice 1 day before IRI; mice were harvested on day 1 after IRI. B. Gating strategies for TIM1^+^ Bregs in the spleen, heart, and blood. C. Immunohistochemical analysis of the level of TIM1 in the sham, IRI, and IRI+RMT1-10 groups.

**Figure S2**

A. The number of Treg cells (CD4^+^ CD25^+^ Foxp3^+^) in the Sham, IRI, and IRI+RMT1‑10 groups were determined in the spleen, heart, and blood using Flow cytometry. B. Flow cytometry analysis of the number of Treg cells (CD4^+^ CD25^+^ Foxp3^+^) in spleen, heart, and blood of three groups (IRI, IRI+RMT1-10, Anti-CD20+IRI+RMT1-10). C. The percent of CD4^+^ and CD8^+^ cells in the different groups, as determined using Flow cytometry.

**Figure S3**

KEGG enriched EGFR signaling pathways

**Figure S4**

The PCA graph was used to demonstrate that the differences between groups are greater than those within groups.
