# Supplementary figures and images for: The effect of TIM1+ Breg cells in myocardial ischemia-reperfusion injury
Source: Cell Death Discov. 2025 Oct 7;11:453. doi: 10.1038/s41420-025-02725-0 (PMC12504465; doi:10.1038/s41420-025-02725-0)

Figure 7

EGFR


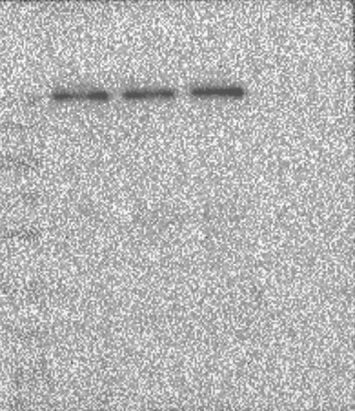


GAPDH


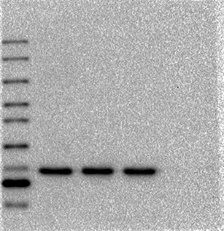


p-EGFR


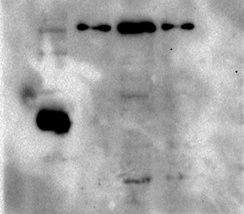


STAT3


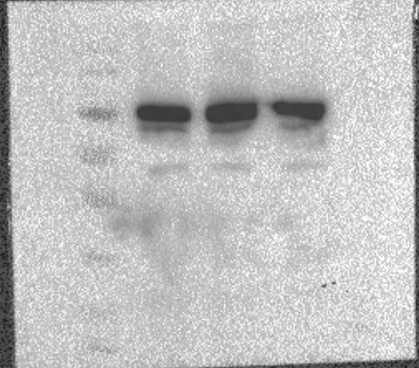


p-STAT3


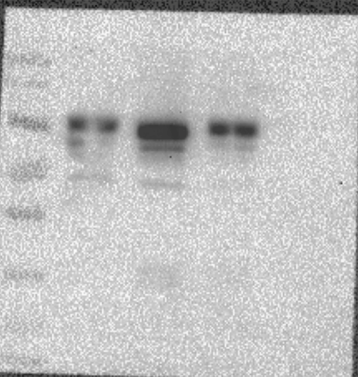

Supplement: Supplementary file 2 — Original data 1 [file 41420_2025_2725_MOESM2_ESM.docx]

AKT1


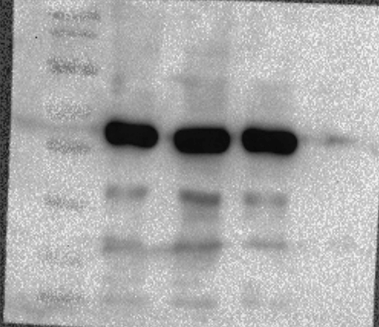


p-AKT1


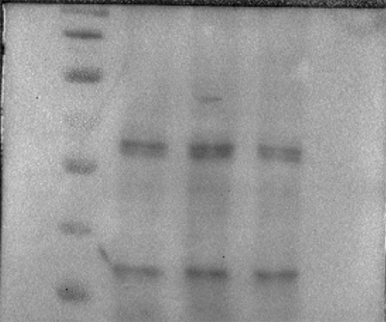


ERK1/2


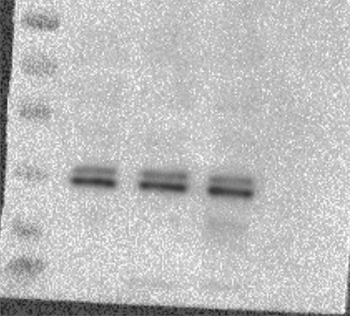


p-ERK1/2


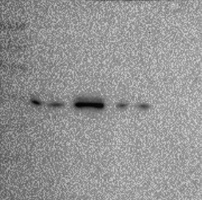


GAPDH


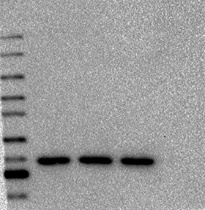

Supplement: Supplementary file 3 — Original data 2 [file 41420_2025_2725_MOESM3_ESM.docx]

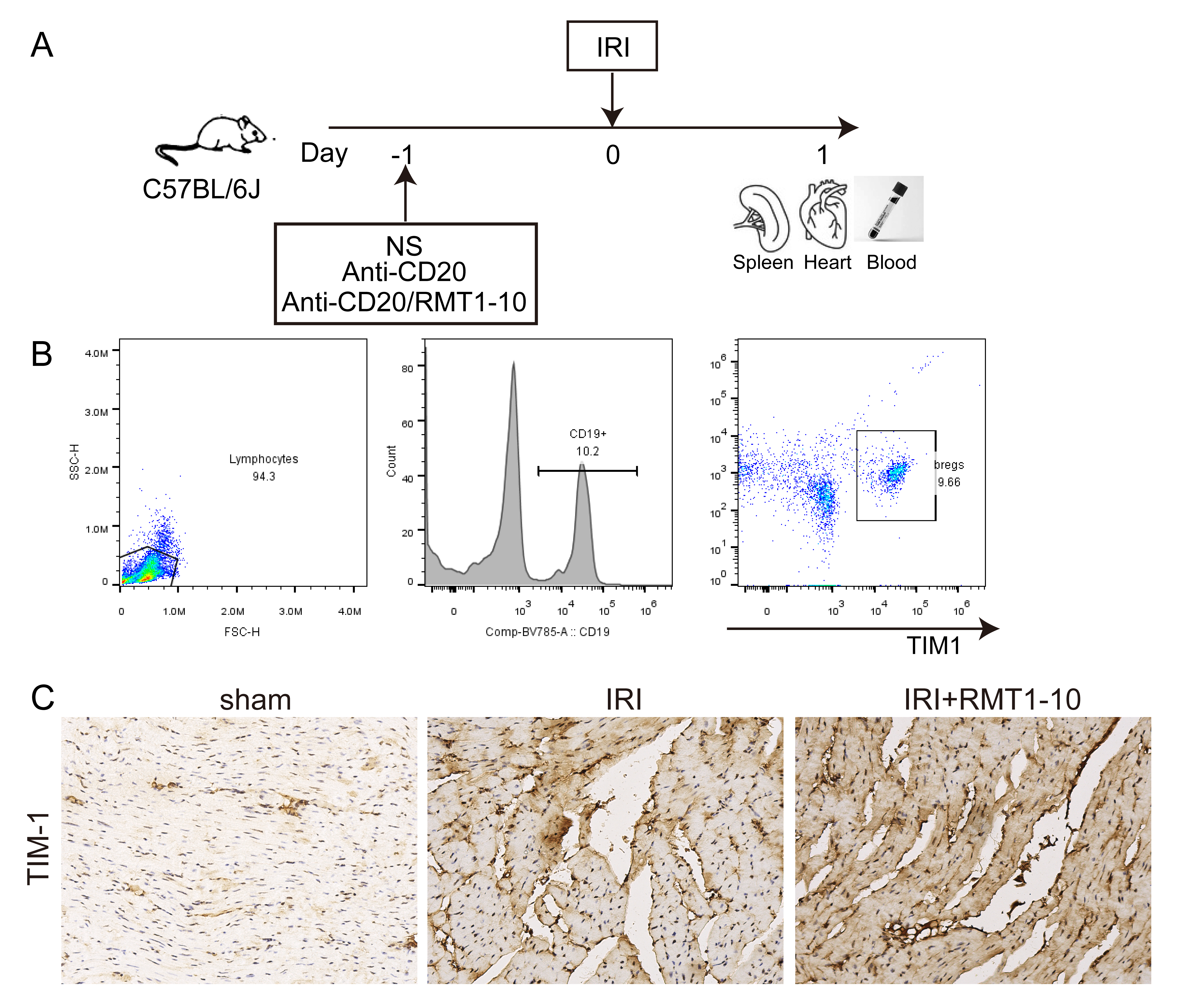

Supplement: Supplementary file 4 — Supplement Figure 1 [file 41420_2025_2725_MOESM4_ESM.tif]

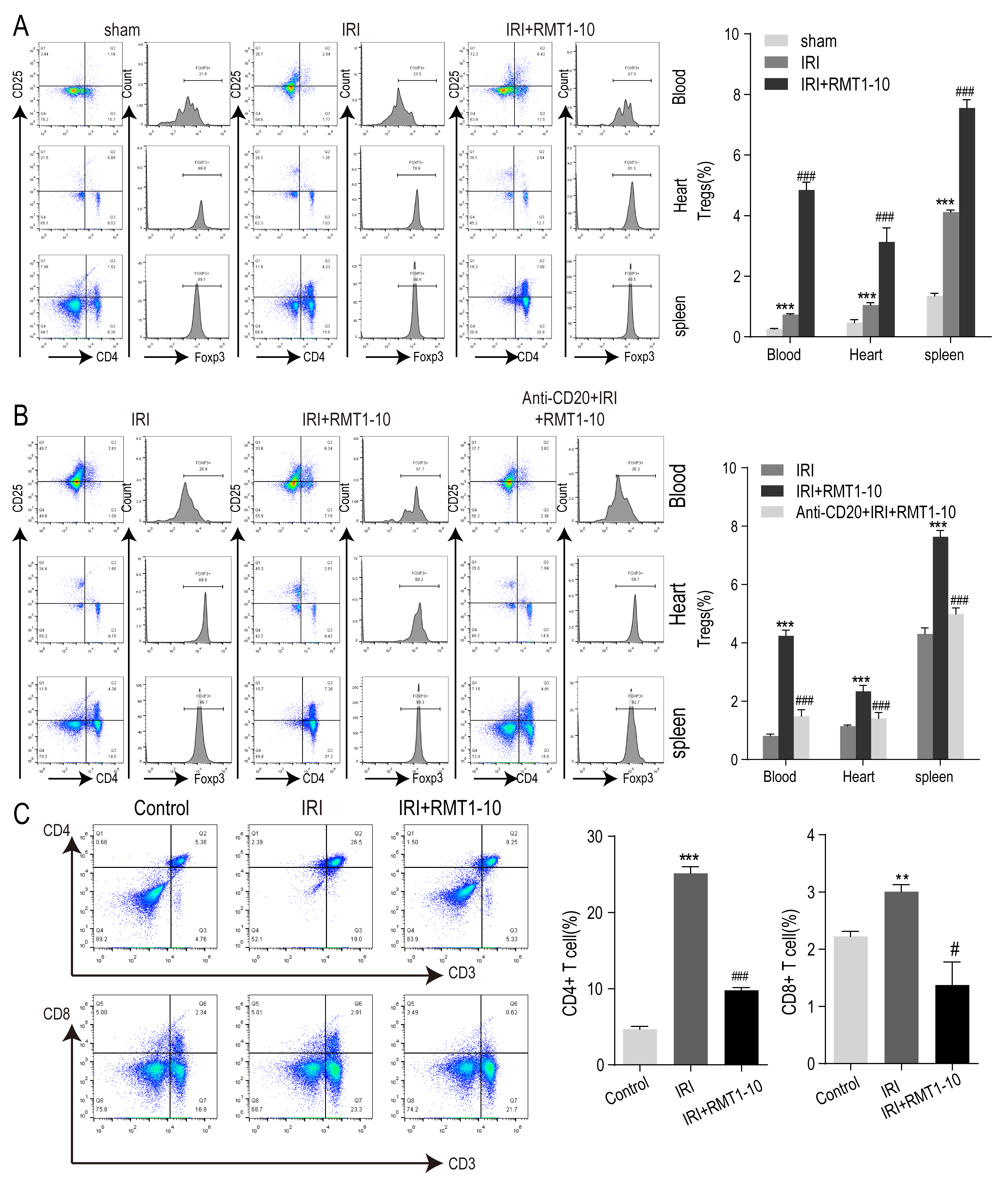

Supplement: Supplementary file 5 — Supplement Figure 2 [file 41420_2025_2725_MOESM5_ESM.tif]

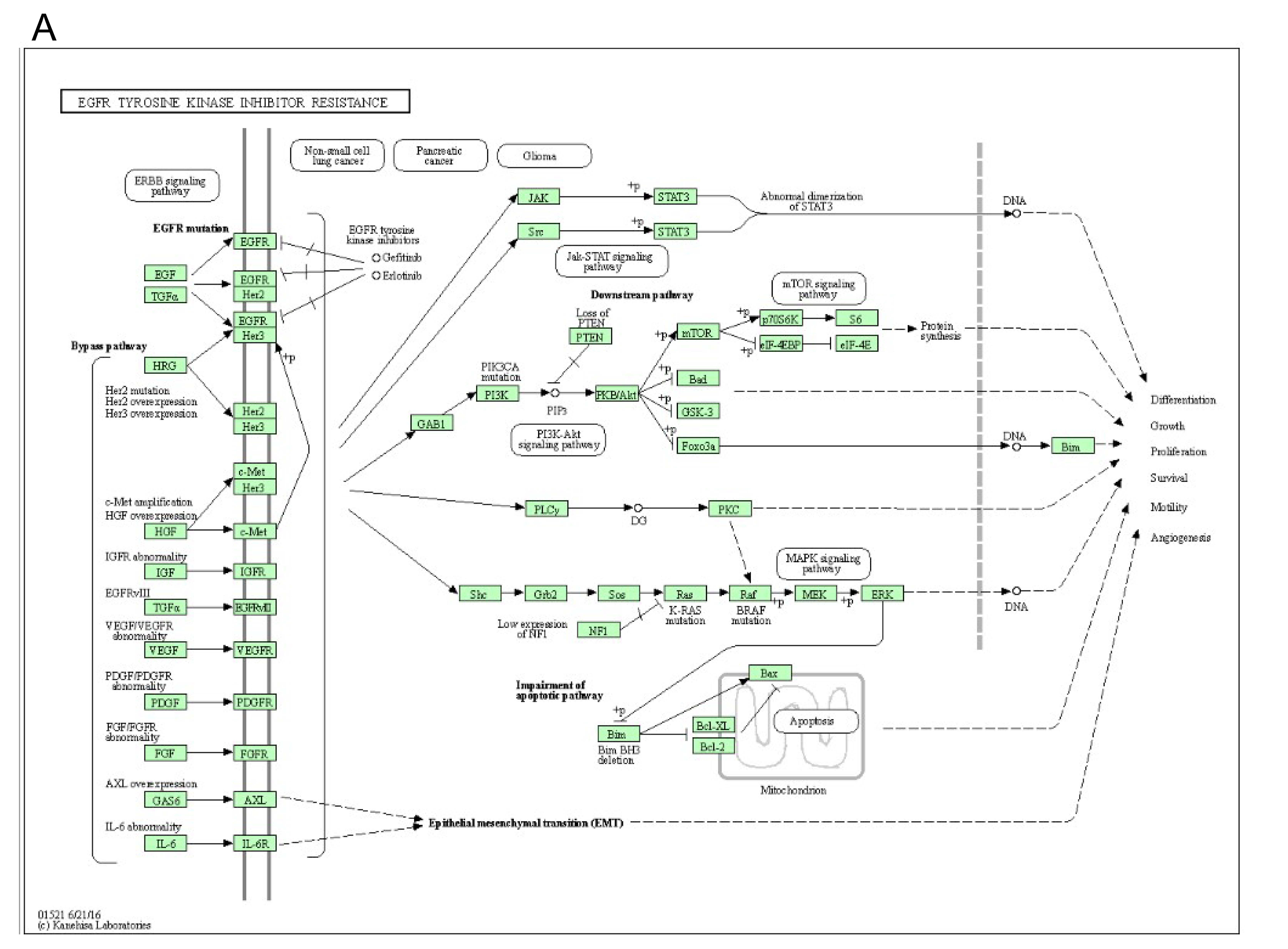

Supplement: Supplementary file 6 — Supplement Figure 3 [file 41420_2025_2725_MOESM6_ESM.tif]

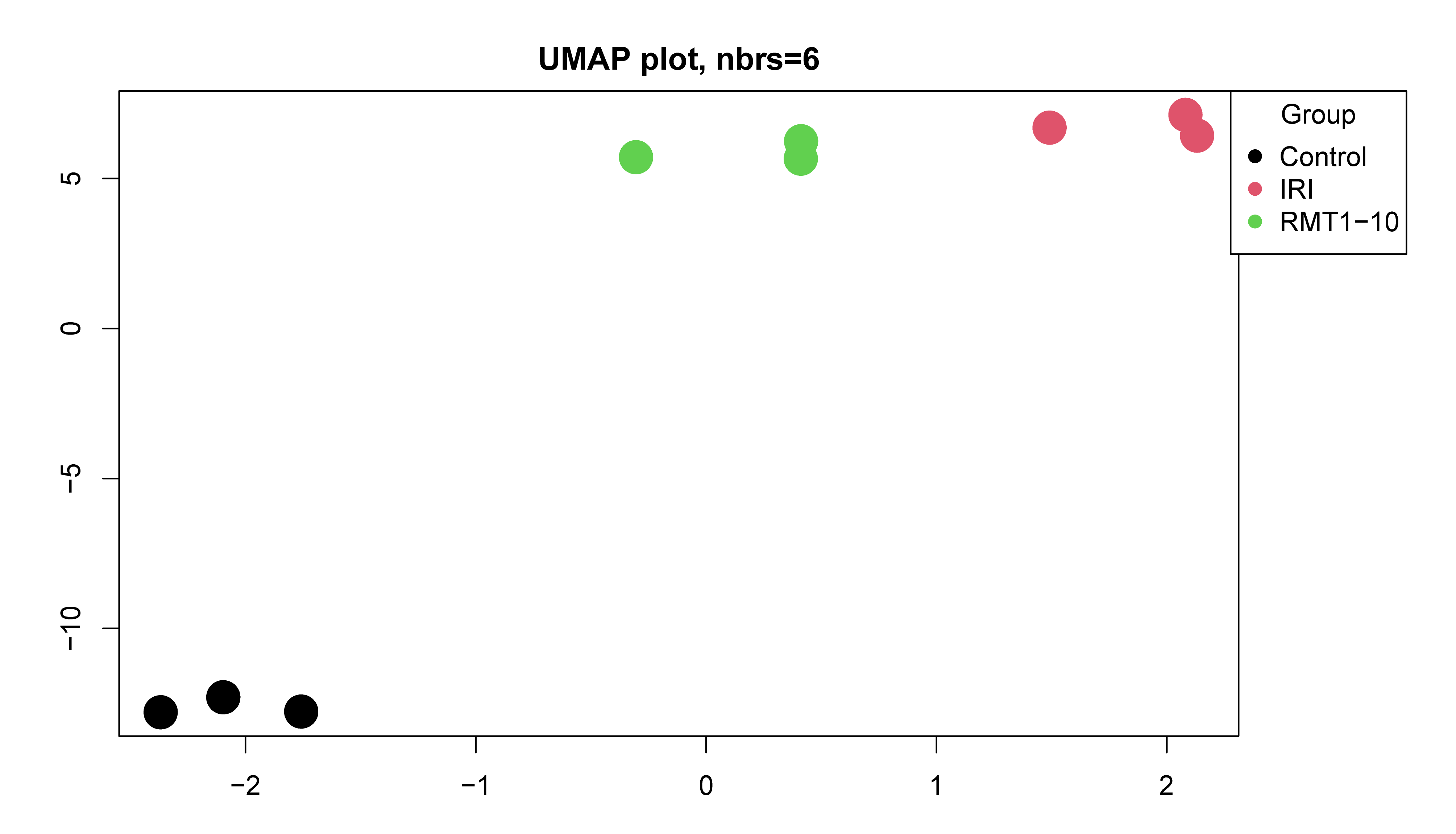

Supplement: Supplementary file 7 — Supplement Figure 4 [file 41420_2025_2725_MOESM7_ESM.tif]
